# Intimate Partner Violence, COVID-19 Stressors, and Intersectionality During the Perinatal Period: Dissecting the Perfect Storm

**DOI:** 10.1177/26884844251380143

**Published:** 2025-09-29

**Authors:** Golfo Tzilos Wernette, Victoria Angenent-Mari, Ananda Sen, Dongru Chen, Kristina Countryman, Dawn M. Johnson, Maria Muzik, Caron Zlotnick

**Affiliations:** ^1^ Department of Family Medicine, University of Michigan Medical School, Ann Arbor, Michigan, USA.; ^2^ Department of Medicine, Women & Infants Hospital of Rhode Island, Providence, Rhode Island, USA.; ^3^ Department of Biostatistics, School of Public Health, University of Michigan, Ann Arbor, Michigan, USA.; ^4^ Department of Psychology, University of Akron, Akron, Ohio, USA.; ^5^ Department of Psychiatry, University of Michigan Medical School, Ann Arbor, Michigan, USA.; ^6^ Department of Obstetrics & Gynecology, University of Michigan Medical School, Ann Arbor, Michigan, USA.; ^7^ Department of Psychiatry and Human Behavior, Brown University Warren Alpert Medical School, Providence, Rhode Island, USA.; ^8^ Department of Psychiatry and Mental Health, University of Cape Town, Rondebosch, South Africa.

**Keywords:** COVID-19, intimate partner violence, mental health, postpartum, pregnancy

## Abstract

**Purpose:**

Intimate partner violence (IPV) and related stressors increased during the COVID-19 pandemic, uniquely impacting perinatal women during a vulnerable time. This study examined the association between IPV, psychosocial correlates, and COVID-19 stressors.

**Methods:**

Our sample included 122 pregnant and postpartum women (average age = 30.1 years; standard deviation = 6.2 years) enrolled in a multisite clinical trial evaluating an IPV-focused intervention for perinatal women who had sought mental health treatment within the last year. Baseline data association between partner abuse (physical, emotional/sexual, severe combined, harassment) and sociodemographic variables was investigated. We analyzed sociodemographic characteristics and measures of Positive Affect and Well Being, Emotional Support, Empowerment, and Self-Efficacy. Furthermore, we conducted an exploratory analysis to examine the role of the intersection between education and employment status on IPV.

**Results:**

Participants reporting more COVID-19-related stress (above median) perceived higher emotional abuse than those in the lower half of the stress spectrum (*p* = 0.04). Partner emotional abuse was inversely associated with Emotional Support (*r* = −0.26, *p* = 0.004) and otherwise not correlated with other psychosocial measures. Perinatal women reporting the most abuse were those reporting part-time employment and an educational level of less than a high school diploma. Ethnicity, pregnancy status, and education were all associated with the severe abuse.

**Conclusion:**

Overall, we demonstrate associations between greater emotional abuse and greater levels of both COVID-19-related stress and lack of emotional support. Also, multiple, overlapping, sociodemographic characteristics impacted perinatal IPV risk. Results offer promising direction for future research.

## Introduction

Intimate partner violence (IPV) has a devastating effect on women’s health and can present in the form of physical (*e.g.*, hitting, kicking, slapping), emotional (or physical threats), or sexual violence.^1^ Though the impacts of IPV can vary within individual cases, IPV has been recognized as one of the major drivers of morbidity and mortality and a significant cause of homicides toward women in particular.^2^ IPV often increases during pregnancy, with estimates showing IPV during pregnancy as high as 20.4%.^3^ IPV during the peripartum period has risks not only for the safety of the pregnant and postpartum individuals but also for neonates. Several literature reviews have examined trends regarding IPV during the perinatal period and found significant neonatal health impacts, including preterm birth, low birth weight, small for gestational age, premature rupture of membranes, miscarriage, and even perinatal death.^4,5^

During the COVID-19 pandemic, starting in 2019 and spreading globally in 2020, IPV was shown to increase. Estimates showed total pooled prevalence of IPV at 22% during the COVID-19 pandemic.^6^ Other studies found a 101% increase in IPV during the first 2 months of the pandemic.^7^ One factor that contributed to the increase in IPV that pregnant individuals experienced during COVID-19 was the unprecedented rise of unstable housing. Unstable or unsafe living situations increased 38% among pregnant women in the first month of the pandemic alone.^7^ Furthermore, the COVID-19 pandemic had significant negative impacts on mental health, with studies finding that individuals experiencing IPV had increased depressive symptoms during the pandemic.^8^

Previous research has demonstrated how interactions between sociodemographic constructs of identity (*e.g.*, race, gender, sexuality, education, socioeconomic status) and systems of power and privilege (where one group of people maintains more resources and advantages than another group) in a society greatly affect different populations’ health outcomes.^9,10^ Known as intersectionality, this theoretical framework provides an important lens for understanding the effects certain social identities can have on the health and well-being of marginalized perinatal women.^11,12^ The principle of intersectionality conceptualizes the interconnected nature of social categories, including gender, race, and class, which contributes to discrimination and inequalities toward groups of people, including women and perinatal women experiencing violence. Including an intersectionality framework when examining data can provide a richer perspective on how these multiple social identities shape the experience of a particular group of people and can help develop equity-informed interventions.

The objective of the current study is to examine associations in baseline data on partner abuse, sociodemographic characteristics, COVID-related stress perception, and personal well-being among a sample of women recruited into our parent study, which was a multisite clinical trial testing the efficacy of an IPV-focused brief, web-based intervention (Strength for U in Relationship Empowerment—SURE) in reducing IPV during the perinatal period.^13^ Specifically, we aim to add to existing literature by examining the associations between women’s experiences of partner abuse and their self-rated experiences of quality of life, emotional support, empowerment, and perceived stress within a unique population of mental health treatment-seeking perinatal women with IPV exposure during the COVID-19 pandemic. Additionally, we novelly examine the intersectionality between the sociodemographic factors of education level and employment status on the perinatal woman’s experience with IPV. We chose to examine these variables, as previous research has shown associations between education level and employment status as independent risk factors for women experiencing IPV. By analyzing the intersection of these two variables, rather than analyzing them independently, we hope to gain a clearer picture of how they shape perinatal IPV risk.

## Materials and Methods

### Procedures

The current article presents an examination of baseline survey correlational data obtained from a parent interventional study. The parent interventional study tested an innovative, web-based intervention, SURE,^13^ for the use with perinatal women exposed to IPV. SURE is a brief, technology-delivered, theory-driven program that incorporates motivational interviewing strategies and empowerment strategies. The parent study was a two-group, randomized, controlled design of a single session plus one telephone booster session within 1 month of completing the web-based intervention session. Recruitment was conducted at two clinical sites in the Midwest and Northeast that provide mental health treatment specifically to perinatal women, including low-income perinatal women.

We collected the baseline survey data with validated measures either in a safe location or by phone through a research assistant. Participants completed the following validated baseline surveys, which were examined in the present article:

1. *Composite Abuse Scale (CAS)*, which measures a wide range of experiences from physical to mental abuse and harassment and is comprised of four subscales: (1) severe combined abuse, (2) physical abuse, (3) emotional/sexual abuse, and (4) harassment.^14^ The CAS is a 30-item inventory where each question seeks to identify the severity of partner abuse on a 6-point response scale ranging from “never” to “daily.” Following strategies of treating missingness detailed in the CAS manual,^15^ we created aggregates of overall CAS scales as well as subscales for physical abuse, emotional abuse, and harassment. Additionally, certain items were combined to generate a severe combined abuse subscale. Presence/absence of specific categories of abuse (physical, emotional, severe combined, harassment) were created analogously. Higher values of the aggregates signify greater degree of perceived abuse in the respective attributes.^14^
2. *Positive Affect and Well Being (QOL) scale*^16^ is measured by using the NIH Neuro-QoL scale (9 items) for positive affect and well-being. The items tap on aspects of a person’s life that relate to a sense of well-being, life satisfaction, or an overall sense of purpose.
3. *Generalized Self-Efficacy (GSE) scale* is a 10-item scale that measures patients’ beliefs in their ability to overcome obstacles and is correlated with emotion, optimism, and satisfaction.^17^
4. *Emotional Support (PROMIS) scale*,^18^ which measures perceived emotional support using a 4-item scale developed by the Patient-Reported Outcomes Measurement Information System (PROMIS).
5. *Personal Progress Scale-Revised (PPS-R)* is a 28-item scale that measures internal sense of empowerment and focuses on key outcomes connected with empowerment, including ability to navigate social systems and positive self-evaluation.^19^
6. *COVID-19 Family Stress Scale*, which was administered to participants who were enrolled during the COVID-19 pandemic.^20^ This scale is a 10-item self-reported questionnaire that taps on different areas of familial stress and hardship caused by the onset of the pandemic (*e.g.*, “Because of COVID-19-related events and changes, I have felt increased stress about less communication with friends and family”) with response options on a 1–5 Likert scale (strongly disagree to strongly agree). Seven of the ten questions had a 5-point Likert response scale, whereas one question had a 3-point response scale. The eight stress questions were aggregated to create a composite COVID score. Higher scores indicated an increasing degree of stress.

To maintain some balance in the data categories, education, marital status, and employment status were recategorized by collapsing original categories. For example, the less than high school diploma and high school diploma/GED were combined into a single category, whereas the tech/trade school and some college categories were combined. Finally, college and postgraduate categories were collapsed to eventually yield a three-category educational status variable. Similarly, marital status was converted to a three-category variable by keeping legally married as its own category, combining separated, divorced, and single without relationship into a single category, and collapsing single with relationship and single with same-sex partner into a category of its own. A three-category employment status variable was further created by giving full-time and part-time statuses their own categories, but collapsing all other categories (housewife, student, retired, and unemployed) into a single category identifying the participants as “not currently employed.” We also created a variable indicating low-income status by dichotomizing at the annual household income of $30,000.

### Participants

Recruitment took place between January 2020 and August 2023. Our study sample was recruited from two study sites (health systems in the Midwest and Northeast) as well as social media recruitment in each of these states. Recruitment from our study sites was conducted in-person (research staff approached women at clinic) and remotely (research staff identified a pool of potential recruits) using electronic health records and contacted them directly using phone/text/email. Table 1 provides a complete summary description of key participant characteristics, both overall and by study site. Study inclusion criteria included: (1) pregnant or up to 12 months postpartum, (2) any IPV during the past 12 months as measured by the Woman Abuse Screening Tool,^21^ (3) ability to understand study procedures in English, (4) between ages of 18 and 45 years old and receiving clinical care at either of the two sites, and (5) reported seeking mental health treatment within the past year. The study protocol was approved by the Institutional Review Board (HUM00166275) and registered on ClinicalTrials.gov (NCT04218864).

**Table 1.** Participant Demographics

Variables	Overall (*N* = 122)	Site		*p* Value
		MI (*N* = 65)	RI (*N* = 57)	
Current age (mean, SD)	30.1, 6.2	30.0, 6.0	30.2, 6.5	0.80
Ethnicity (*N*, %)				**0.03**
Hispanic	25, 20.5	8, 12.3	17, 29.8	
Non-Hispanic	97, 79.5	57, 87.7	40, 70.2	
Race (*N*, %)				**0.03**
Caucasian/White	78, 63.9	46, 70.8	32, 56.1	
Black	29, 23.8	15, 23.1	14, 24.6	
Asian	1, 0.8	1, 1.5	0, 0.0	
Native American or Native Alaskan	2, 1.6	0, 0.0	2, 3.5	
Multiple races or ethnicities	6, 4.9	3, 4.6	3, 5.3	
Other	6, 4.9	0, 0.0	6, 10.5	
Marital status (*N*, %)				**0.02**
Married	36, 29.8	27, 41.5	9, 16.1	
Separated	7, 5.8	2, 3.1	5, 8.9	
Divorced	6, 5.0	3, 4.6	3, 5.4	
Single, no relationship	34, 28.1	17, 26.2	17, 30.4	
Single, in a relationship	37, 30.6	15, 23.1	22, 39.3	
Single, same sex partner	1, 0.8	1, 1.5	0, 0.0	
Employment status (*N*, %)				**0.01**
Full time	49, 40.5	32, 49.2	17, 30.4	
Part time	18, 14.9	7, 10.8	11, 19.6	
Student	6, 5.0	4, 6.2	2, 3.6	
Housewife	15, 12.4	11, 16.9	4, 7.1	
Unemployed	33, 27.3	11, 16.9	22, 39.3	
Education (*N*, %)				**0.03**
Less than HS	3, 2.5	2, 3.1	1, 1.8	
HS/GED	23, 19.0	7, 10.8	16, 28.6	
Tech/trade school	5, 4.1	2, 3.1	3, 5.4	
Some colleges	39, 32.2	19, 29.2	20, 35.7	
College graduate	25, 20.7	15, 23.1	10, 17.9	
Postgraduate	26, 21.5	20, 30.8	6, 10.7	
No. children (*N*, %)				0.40
0	13, 10.7	7, 10.8	6, 10.7	
1	47, 38.8	29, 44.6	18, 32.1	
2	33, 27.3	14, 21.5	19, 33.9	
3 or more	28, 23.1	15, 23.1	13, 23.2	
No. adults (*N*, %)				**0.01**
1	43, 35.5	20, 30.8	23, 41.1	
2	49, 40.5	34, 52.3	15, 26.8	
3 or more	29, 24.0	11, 16.9	18, 32.1	
Pregnant (*N*, %)				0.06
No	91, 75.2	44, 67.7	47, 83.9	
Yes	30, 24.8	21, 32.3	9, 16.1	
Current partner (*N*, %)				0.20
No	46, 38.0	21, 32.3	25, 44.6	
Yes	75, 62.0	44, 67.7	31, 55.4	
Arm allocation (*N*, %)				0.40
Intervention	65, 53.3	37, 56.9	28, 49.1	
Control	57, 46.7	28, 43.1	29, 50.9	

Bold text signifies statistical significance.

SD, standard deviation.

### Data analytic plan

Responses on several IPV items were collected. These responses were aggregated to yield summary measures that were analyzed. The tools and measures are described below. The interrelationship between the CAS scores and secondary outcome measures (QOL, GSE, PPS-R, PROMIS) was explored through Pearson correlation coefficient. Multivariable linear regression models were explored to identify associations between the CAS scores and sociodemographic variables. Final selection of demographic variables to be included in the regression model was guided by their univariate associations with the outcome that were significant at *p* < 0.15.

Intersectional analysis is becoming increasingly popular, which tends to view the outcome experienced by an individual as a function of the collective effect of factors rather than marginal effects of the individual factors. In this vein, we carried out exploratory analysis by considering the nine cross-classified categories created by combining education and employment status. The regression analysis was then repeated using these categories.

## Results

Data were collected on 122 pregnant and postpartum women across two sites, in the Midwest (*n* = 65) and in the Northeast (*n* = 57). Participants in the Midwest site had a slightly higher proportion of married, pregnant, and White pregnant women in their participant pool than the site in the Northeast. By contrast, the Northeast site had a significantly higher percentage of Hispanic, as well as unemployed, pregnant women. All baseline outcome measures were missing for one subject due to a software issue; therefore, outcome analysis was carried out with data from 121 participants (Table 1).

In bivariate correlations, CAS variables were generally not highly correlated with outcome measures except for the Emotional Support scale (PROMIS). The PROMIS total was most correlated with the Emotional Abuse subscale (*r* = −0.26, *p* = 0.004), such that women reporting higher levels of emotional abuse reported the least emotional support. PROMIS was also correlated with harassment (*r* = −0.19, *p* = 0.04) and total CAS (*r* = −0.21, *p* = 0.02). The Empowerment total scale was mildly correlated with the Emotional Abuse subscale (*r* = −0.18, *p* = 0.05) and the Severe Combined Abuse subscale (*r* = −0.17, *p* = 0.06).

### Association between CAS scores and sociodemographic variables

Using multivariable regression models, we explored the unique contribution of each sociodemographic indicator, that is, age, ethnicity, partner status, pregnancy/postpartum status, marital status, education, employment, and site as predictors of CAS scores (overall and subscales). Some significant associations with demographic variables were observed (Table 2). Perinatal women and women with a current partner experienced significantly less overall abuse than their counterparts based on mean CAS scores (*p* = 0.01, *p* = 0.05, respectively). Individuals with a college/postgrad degree felt less abused overall compared with the individuals whose education level was less than or equal to high school diploma/GED (*p* = 0.004), and those with a tech/trade degree and some college, respectively (*p* = 0.0004). Similar associations were obtained between the Emotional Abuse subscale and education. Individuals with a college/postgrad degree experienced significantly lower emotional abuse compared with the participants whose education level was less than or equal to high school diploma/GED, as well as those with a tech/trade degree and some college. On the other hand, individuals who were employed full-time perceived the highest degree of emotional abuse. In fact, they scored significantly higher compared with the “not currently employed” pool of women (*p* = 0.02), while the difference between full- and part-time individuals did not cross the significance threshold.

**Table 2.** Association Between CAS and Sociodemographic Characteristics

Regression results	CAS total			Emotional abuse subscale			Physical abuse subscale			Severe combined abuse subscale			Harassment subscale		
	Estimate	SE	*p* Value	Estimate	SE	*p* Value	Estimate	SE	*p* Value	Estimate	SE	*p* Value	Estimate	SE	*p* Value
Age at enrollment	0.167	0.35	0.637	0.2	0.2	0.37	−0.06	0.1	0.53	0.02	0.05	0.71	0.01	0.07	0.8413
Ethnicity (ref: Hispanic/Latina)	8.03	4.64	0.086	−1.01	2.9	0.72	−4.75	1.26	**0.0003**	−2.37	0.69	**0.0008**	0.14	0.92	0.8778
Current partner status (ref: No)	−14.9	7.51	**0.05**	−6.59	4.63	0.16	−5.23	2.04	**0.01**	−1.71	1.12	0.13	−1.43	1.48	0.3374
Pregnancy status (ref: No)	−11.55	4.42	**0.01**	−4.17	2.72	0.13	−4.35	1.2	**0.0004**	−1.72	0.66	**0.0102**	−1.33	0.87	0.1288
Education (ref: Grad/postgrad)															
Less than HS	16.95	5.76	**0.01**	10.04	3.55	**0.006**	1.67	1.56	0.29	2.16	0.86	**0.0133**	3.03	1.14	**0.0088**
Tech/trade/some college	17.35	4.75	**0.0004**	10.5	2.9	**0.001**	2.65	1.29	**0.04**	1.28	0.7	0.07	2.87	0.94	**0.0028**
Employment status (ref: Full time)															
Part time	0.73	5.35	0.891	−1.09	3.3	0.74	1.7	1.45	0.25	1.14	0.8	0.15	−1.08	1.06	0.3081
Not employed	−6.36	4.08	0.122	−5.74	2.5	**0.025**	1.66	1.11	0.14	0.39	0.6	0.52	−2.72	0.8	**0.001**
Marital status (ref: Currently single)															
Currently married	0.97	7.76	0.9006	−1.12	4.8	0.82	2.33	2.11	0.27	1.19	1.15	0.31	−1.46	1.53	0.3416
Single in relationship	4.14	7.82	0.5978	0.12	4.8	0.98	4.28	2.1	**0.05**	0.56	1.16	0.63	−0.85	1.54	0.5847
Site (ref: RI)	2.04	4.01	0.612	−0.51	2.5	0.83	2.42	1.09	**0.03**	0.64	0.6	0.29	−0.56	0.79	0.4793

Bold text signifies statistical significance.

CAS, Composite Abuse Scale; SE, standard error.

Several significant associations existed between the physical abuse scores and the independent variables. Non-Hispanic women, pregnant women, and women with a current partner felt a significantly lower degree of physical abuse than their counterparts (*p* = 0.0003, *p* = 0.0004, *p* = 0.01, respectively). Women who went to tech/trade school or had some college had experienced significantly higher degree of physical abuse than those with a college/postgraduate degree (*p* = 0.04). Unmarried women in a relationship scored mildly significantly higher on the perceived physical abuse scale compared with the single women in no relationship (*p* = 0.05). Even after adjusting for demographic heterogeneity in the participant pool, subjects recruited from the MI site reported more physical abuse than their peers at the RI site (*p* = 0.03).

Ethnicity, pregnancy status, and education were all associated with the severe combined abuse subscale. The directionality was similar for the other subscales, with Hispanic/Latina women and perinatal women scoring significantly higher than their respective comparators (*p* = 0.0008, *p* = 0.01, respectively). Women with a college/postgrad degree perceived significantly lower severe combined abuse compared with the pregnant women whose education level was less than or equal to high school diploma/GED (*p* = 0.01). Education level was again strongly associated with the perception of harassment. Women with a college/postgraduate degree felt less harassed than either pregnant women with an education level less than or equal to a high school diploma/GED (*p* = 0.009) or those who went to tech/trade school or had some college (*p* = 0.003). In addition, women who were employed full time perceived higher level of harassment compared with the “not currently employed” pool of women (*p* = 0.001).

### Association between sociodemographic variables and variables of personal well-being

Substantially fewer sociodemographic characteristics were associated with the secondary outcomes of women’s well-being (Table 3). Based on the patient-reported measure, women with a current partner exhibited a higher level of emotional support than those who did not have one (*p* = 0.01). Women who reported single status had a higher PROMIS score on average compared with those who were currently married (*p* = 0.0075), as well as those who were single but in a relationship (*p* = 0.05). Not surprisingly, women with a college or postgraduate degree felt a higher level of empowerment compared with those with less than or equal to a high school diploma/GED (*p* = 0.036). No other sociodemographic association was observed among these or other outcome measures.

**Table 3.** Association Between the Secondary Outcomes and Sociodemographic Characteristics

Variables	QOL			PROMIS			PPS-R			GSE		
	Estimate	SE	*p* Value	Estimate	SE	*p* Value	Estimate	SE	*p* Value	Estimate	SE	*p* Value
Age at enrollment	−0.0463	0.1435	0.7476	−0.0071	0.0789	0.9287	−0.7528	0.4178	0.0743	−0.1338	0.0897	0.1388
Ethnicity (ref: Hispanic/Latina)	0.2302	1.8813	0.9028	0.4107	1.0338	0.6919	3.1236	5.4774	0.5697	0.4202	1.1760	0.7216
Current partner status (ref: No)	0.4230	3.0461	0.8898	4.3444	1.6738	**0.0107**	−1.1616	8.8687	0.8960	1.5207	1.9041	0.4262
Pregnancy status (ref: No)	0.3506	1.7906	0.8451	1.0359	0.9839	0.2947	−2.0958	5.2133	0.6885	−0.8339	1.1193	0.4579
Education (ref: Graduate/postgraduate)												
Less than HS	−2.2737	2.3333	0.3320	−1.1645	1.2822	0.3657	−14.4027	6.7935	**0.0363**	−2.1568	1.4585	0.1421
Tech/trade/some college	−0.0418	1.9255	0.9827	−1.5320	1.0581	0.1505	−9.4560	5.6061	0.0945	−0.5279	1.2036	0.6618
Employment status (ref: Full time)												
Part time	0.7064	2.1696	0.7454	0.7085	1.1922	0.5535	−0.9418	6.3167	0.8818	−1.4413	1.3562	0.2902
Not employed	0.3709	1.6552	0.8231	−0.1075	0.9095	0.9061	−5.2319	4.8192	0.2800	−0.6160	1.0347	0.5528
Marital status (ref: Currently single)												
Currently married	−2.1682	3.1469	0.4923	−4.7147	1.7292	**0.0075**	−9.6625	9.1621	0.2939	−3.1397	1.9671	0.1134
Single in relationship	−0.9902	3.1686	0.7553	−3.4254	1.7411	0.0517	−2.5244	9.2255	0.7849	−3.1366	1.9807	0.1162
Site (ref: RI)	0.4455	1.6266	0.7847	0.6688	0.8938	0.4559	0.7487	4.7359	0.8747	0.3627	1.0168	0.7220

Bold text signifies statistical significance.

GSE, Generalized Self-Efficacy; PPS-R, Personal Progress Scale-revised; PROMIS, Patient-Reported Outcomes Measurement Information System; SE, standard error.

### Association between COVID-19 stress variables and variables of personal well-being

The COVID-19 stress survey responses were available for 113 participants, as the survey was only introduced after a period of recruitment during the COVID pandemic. The regression models adjusted for the dichotomous COVID score showed virtually identical patterns of association to the models without the COVID-19 variables, with some of the earlier borderline associations becoming nonsignificant (data not shown).

### Intersectionality analysis

**FIG. 1.**
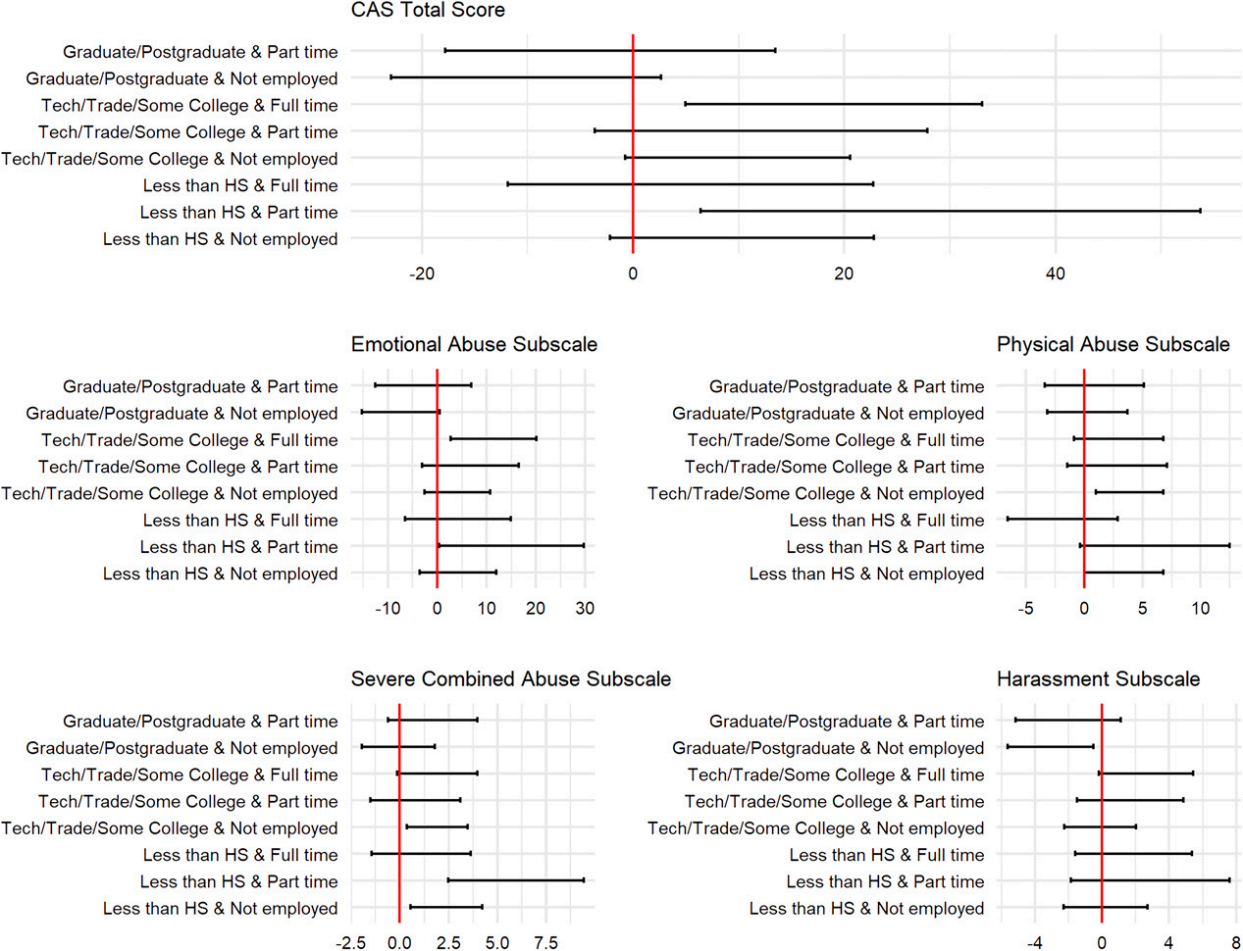
Pairwise differences between the intersection categories for CAS scales (ref: Graduate/postgraduate and full time). CAS, Composite Abuse Scale.

**FIG. 2.**
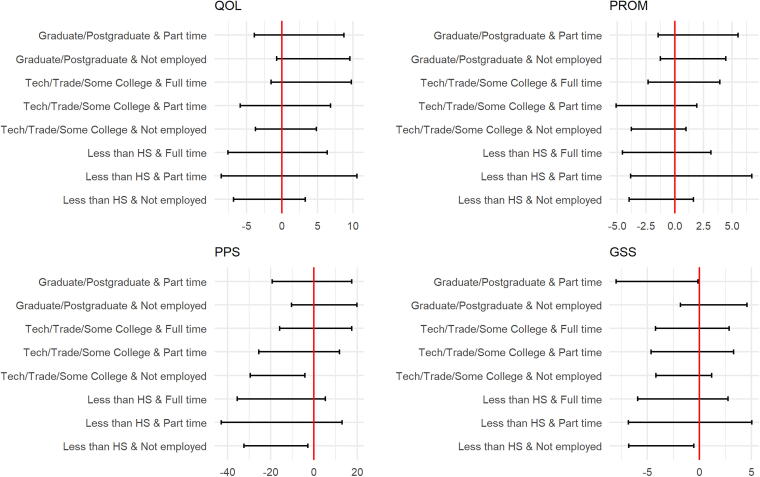
Differences of intersection categories from graduate/postgraduate and full-time category for the secondary outcomes.

As explained in the methods section, an alternative analysis involving a cross-classification of the categorical representation of education and employment was carried out. Table 4 shows the distribution across these categories. For reference, we chose the most privileged category of women who had a graduate/postgraduate degree and were full-time employed. The forest plots in Figures 1 and 2 demonstrate the average estimated difference (with associated 95% confidence intervals) in outcome values between the reference and the other intersection categories. Instead of overwhelming the readers by reporting all 36 pairwise differences, we chose to present representative differences that exhibit the general trend. The intervals that stay either entirely to the right or left of the vertical line at zero demonstrate statistical significance at 5% level. For all CAS scales and subscales, the women who were employed part-time coupled with the educational status of less than high school consistently turned out to be the most abused (Fig. 1). Among the other classifications, full- or part-time employees with a tech/trade/some college degree scored high across the different CAS subscales. On the other extreme, the least abused category was generally comprised of women who were unemployed with a college/postgraduate degree.

**Table 4.** Distribution Across Cross-Classified Education and Employment Categories

Education	Employment status	Frequency *N* = 121	%
Less than HS	Full time	7	5.79
	Part time	3	2.48
	Not employed	16	13.22
Tech/trade/some college	Full time	10	8.26
	Part time	8	6.61
	Not employed	26	21.49
Graduate/postgraduate	Full time	32	26.45
	Part time	7	5.79
	Not employed	12	9.92

Within the secondary outcomes, a clear trend is evident among the unemployed women. Within this subgroup, college-educated women in general felt the most empowered, self-efficacious, and seemed to perceive the most emotional support and score the highest on the QOL scale, followed by women with tech/trade/some college degree, with the women with less than a high school diploma feeling the least empowered (Fig. 2). Most of these differences were not statistically significant, however. It appears that for the Empowerment Score (PPS-R), irrespective of the employment status, women with less than a high school diploma felt less empowered compared with women with a college/postgraduate degree. Among the college graduates, part-time employees had a statistically significantly lower level of self-efficacy compared with full- or part-time employees. Such intersectional insights were not possible to glean from a main-effects-only model.

## Discussion

This study, which only focused on the baseline data from a larger RCT (parent study), found significant associations between the Emotional Support and the CAS Emotional Abuse subscale, which revealed that perinatal women experiencing higher levels of emotional abuse also had the least amount of emotional support. Similarly, analysis of the COVID-19 stressors found that the women reporting the highest COVID-19-related stress were experiencing more emotional abuse than those reporting lower stress. In terms of sociodemographic characteristics, education, employment status, pregnancy status, and ethnicity were the characteristics most associated with the abuse measures. We did not find significant associations with age, race, or income level. Using an intersectional approach to further understand sociodemographic characteristics’ effect on perinatal IPV risk, we found that women with the least amount of education and a part-time employment status experienced the highest IPV levels.

Prior research has also found strong associations between IPV and a lack of emotional support, with one study finding that women experiencing IPV were more than two times as likely to report lower emotional support than women not reporting IPV.^22^ Including pregnant women specifically, Kita et al. found significantly lower emotional support scores in abused pregnant women compared with non-abused pregnant women.^23^ In terms of abuse type during pregnancy, a cross-sectional study conducted in Belgium found that psychological (emotional) abuse was the most common form of IPV during pregnancy, which mirrors the current study findings.^24^ Furthermore, a 2021 narrative review of 86 perinatal IPV studies conducted worldwide found that the most prevalent form of perinatal IPV was psychological/emotional.^25^ A longitudinal study of IPV experienced by women before, during, and after pregnancy revealed that women experiencing a greater severity of IPV during pregnancy were significantly more likely to have poor social support.^26^ Thus, emotional support appears to play a significant role in the lives of perinatal women with IPV. Future research should examine if emotional support is salient to abused women relative to other forms of support. Also, prospective studies should help determine if lack of emotional support is a risk factor for IPV and/or if the presence of IPV restricts access to emotional support.

Emotional abuse was also correlated with stress brought on by the COVID-19 pandemic. Perinatal women reporting more COVID-19 stress had higher emotional abuse scores than those reporting less COVID-19 stress. This may indicate that the pandemic further compounded the stress felt by perinatal women experiencing high levels of emotional abuse, and it may have also increased emotional abuse. Empirical support for this theory exists. An online IPV survey conducted in Michigan during the COVID-19 pandemic found that for those experiencing IPV before the pandemic, the severity of IPV increased during the height of lockdowns during the pandemic.^27^ Furthermore, certain groups experienced significantly higher prevalence of new or increased IPV, and this included pregnant women and women with a toddler at home.^27^ A systematic review of IPV studies conducted in the United States during the COVID-19 pandemic also demonstrated that both IPV frequency and severity exponentially increased after the onset of the pandemic.^28^ While our study did not assess whether the levels of IPV increased or decreased during the COVID-19 pandemic, our finding of higher COVID-19-related stress among those women experiencing the most emotional abuse supports these previous findings.

We found that perinatal women with the lowest education levels and with part- or full-time employment scored the highest across the CAS subscales. Based on mean CAS score, perinatal women with the highest levels of education and reported having a current partner experienced significantly less abuse. Our findings regarding education level are in line with previous perinatal IPV research, with several studies finding that women with lower levels of education experienced more abuse than those with higher levels of educational attainment.^24,25^ Interestingly, we found that pregnant women had lower mean CAS scores than postpartum women, after adjusting for all other variables. Several studies have demonstrated higher levels of IPV^3,25^ during pregnancy compared with other times in a woman’s life, while others have not.^26,29^ This should be interpreted with caution, however, as levels of IPV during pregnancy vary heavily by world region and by the type of IPV being perpetrated.^3^ When addressing IPV during the perinatal period, we need to be cognizant of the type of abuse as well as the cultures and communities that encompass the women.

Since sociodemographic characteristics are inherently linked and overlapping, using an intersectional approach to analysis can present a clearer picture of the results. We found that the perinatal women reporting higher levels of abuse were consistently those reporting part-time employment and an educational level of less than a high school diploma. This finding is not surprising, as decades of IPV research have demonstrated a link between low sociodemographic status (SES) and an increased risk of experiencing IPV.^30^ To be clear, IPV is a phenomenon that transcends all sociodemographic backgrounds and characteristics; individuals of any SES status can experience IPV. There has, however, been a consistent association between having a lower income level and being at higher IPV risk, regardless of racial/ethnic background.^30,31^ However, SES is comprised of more than just income; it also includes education level, employment status, and job type. Findings on education level and employment status have not been as consistent of an IPV predictor as income level, but there are several studies that have shown strong links between lower education and employment levels and higher IPV risk.^30–32^ Even in the current study, the mean CAS scores were associated with education level, but not with employment or low-income status in our standard regression model. It was only when looking at the intersection of these variables that we found women with part-time employment and less than a high school diploma had the highest mean CAS scores, indicating that unless we use an intersectional approach to understand the multiple overlapping layers of SES, we cannot identify who might be most vulnerable to IPV due to circumstances. This finding is consistent with the work of others who have advocated for a more intersectional approach to understand the role of SES on IPV during the perinatal period.^25,31^

Strengths of our study included recruitment at two sites in different states, which provides geographical diversity. Additionally, our protocol was technology-delivered, with additional safety protocols in place for those who were at greater safety risk and wanted to come to the hospital, which may have facilitated disclosure.^33^ While this study has shed light on sociodemographic factors associated with IPV in perinatal women with existing mental health concerns, our sample is treatment-seeking and from two regions of the United States and may not generalize to all pregnant and postpartum women.

## Conclusion

Overall, this study examining baseline survey data from a larger parent study contributes to the literature by examining associations between IPV, psychosocial correlates, and COVID-19 stressors among perinatal women seeking mental health treatment. Additionally, we extend the impact of our findings by examining the role of intersection between education and employment status on IPV.

This work has clinical and societal implications. First, our results provide a useful lens through which to examine and tailor screening and intervention approaches for this vulnerable population of perinatal women. Second, it is imperative to consider the social inequities that subsets of women face to inform culturally relevant interventions for IPV. For example, IPV interventions that also address social inequities experienced by the women (*i.e.*, lack of stable housing, income/job security, educational opportunities) will likely have the most impact.

## Abbreviations Used

CAS: Composite Abuse Scale
GSE: Generalized Self-Efficacy
IPV: Intimate partner violence
PPS-R: Personal Progress Scale-Revised
PROMIS: Patient-Reported Outcomes Measurement Information System
SURE: Strength for U in Relationship Empowerment

## References

[1] American College of Obstetricians and Gynecologists. ACOG Committee Opinion No. 518: Intimate partner violence. Obstet Gynecol 2022;119(2 Pt 1):412–417. 10.1097/AOG.0b013e318249ff74 22270317

[2] Gomez-Casillas A, Lozano M, Rentería E. Expected years lived with intimate partner violence: A new approach for public health. Glob Health Action 2021;14(1):1976442; doi: 10.1080/16549716.2021.1976442 34542024 PMC8462847

[3] Román-Gálvez RM, Martín-Peláez S, Fernández-Félix BM, et al. Worldwide prevalence of intimate partner violence in pregnancy. A systematic review and meta-analysis. Front Public Health 2021;9:738459. 34527656 10.3389/fpubh.2021.738459 PMC8435609

[4] Alhusen JL, Ray E, Sharps P, et al. Intimate partner violence during pregnancy: Maternal and neonatal outcomes. J Womens Health (Larchmt) 2015;24(1):100–106. 25265285 10.1089/jwh.2014.4872 PMC4361157

[5] Pastor-Moreno G, Ruiz-Pérez I, Henares-Montiel J, et al. Intimate partner violence and perinatal health: A systematic review. BJOG 2020;127(5):537–547; doi: 10.1111/1471-0528.16084 31912613

[6] Huldani H, Kamal Abdelbasset W, Abdalkareem Jasim S, et al. Intimate partner violence against pregnant women during the COVID-19 pandemic: A systematic review and meta-analysis. Women Health 2022;62(6):556–564. 35791678 10.1080/03630242.2022.2096755

[7] Avalos LA, Ray GT, Alexeeff SE, et al. Association of the COVID-19 pandemic with unstable and/or unsafe living situations and intimate partner violence among pregnant individuals. JAMA Netw Open 2023;6(2):e230172; doi: 10.1001/jamanetworkopen.2023.0172 36811863 PMC9947729

[8] FitzPatrick KM, Brown SJ, Hegarty K, et al. Experiences of physical and emotional intimate partner violence during the COVID-19 pandemic: A comparison of prepandemic and pandemic data in a longitudinal study of Australian mothers. BMJ Open 2024;14(4):e081382; doi: 10.1136/bmjopen-2023-081382 PMC11056622 38643001

[9] Homan P, Brown TH, King B. Structural intersectionality as a new direction for health disparities research. J Health Soc Behav 2021;62(3):350–370. 34355603 10.1177/00221465211032947 PMC8628816

[10] Heard E, Fitzgerald L, Wigginton B, et al. Applying intersectionality theory in health promotion research and practice. Health Promot Int 2020;35(4):866–876. 31390472 10.1093/heapro/daz080

[11] Crenshaw K. Demarginalizing the intersection of race and sex: A black feminist critique of antidiscrimination doctrine, feminist theory and antiracist politics. Univ Chic Leg Forum 1989;139.

[12] Lapalme J, Haines-Saah R, Frohlich KL. More than a buzzword: How intersectionality can advance social inequalities in health research. Crit Public Health 2020;30(4):494–500.

[13] Johnson DM, Tzilos Wernette G, Miller TR, et al. Computerized intervention for reducing intimate partner victimization for perinatal women seeking mental health treatment: A multisite randomized clinical trial protocol. Contemp Clin Trials 2020;93:106011; doi: 10.1016/j.cct.2020.106011 32305456 PMC7254924

[14] Hegarty K, Sheehan M, Schonfeld C. A multidimensional definition of partner abuse: Development and preliminary validation of the Composite Abuse Scale. Domestic Violence 2017:15–31.

[15] Hegarty K, Valpied J. Composite abuse scale manual. Melbourne: Department of General Practice, University of Melbourne 2007.

[16] Cella D, Nowinski C, Peterman A, et al. The neurology quality-of-life measurement initiative. Arch Phys Med Rehabil 2011;92(10 Suppl):S28–S36; doi: 10.1016/j.apmr.2011.01.025 21958920 PMC3193028

[17] Schwarzer R, Jerusalem M, Weinman J, et al. Measures in health psychology: A user’s portfolio. Causal and control beliefs. NFER-NELSON: Windsor; 1995.

[18] Pilkonis PA, Choi SW, Reise SP, et al.; PROMIS Cooperative Group. Item banks for measuring emotional distress from the Patient-Reported Outcomes Measurement Information System (PROMIS®): Depression, anxiety, and anger. Assessment 2011;18(3):263–283; doi: 10.1177/1073191111411667 21697139 PMC3153635

[19] Johnson DM, Worell J, Chandler RK. Assessing psychological health and empowerment in women: The Personal Progress Scale Revised. Women Health 2005;41(1):109–129; doi: 10.1300/J013v41n01_07 16048871

[20] Huth-Bocks A. COVID-19 family stress screener. SRCD commons; 2020.

[21] Brown JB, Lent B, Schmidt G, et al. Application of the Woman Abuse Screening Tool (WAST) and WAST-short in the family practice setting. J Fam Pract 2000;49(10):896–903. 11052161

[22] Hui V, Constantino RE. The association between life satisfaction, emotional support, and perceived health among women who experienced intimate Partner violence (IPV) - 2007 behavioral risk factor surveillance system. BMC Public Health 2021;21(1):641; doi: 10.1186/s12889-021-10665-4 33794819 PMC8015742

[23] Kita S, Haruna M, Matsuzaki M, et al. Does antenatal social support affect the relationships between intimate partner violence during pregnancy and perinatal mental health? Violence Against Women 2020;26(6–7):573–589; doi: 10.1177/1077801219835052 30940004

[24] Van Parys AS, Deschepper E, Michielsen K, et al. Prevalence and evolution of intimate partner violence before and during pregnancy: A cross-sectional study. BMC Pregnancy Childbirth 2014;14:294; doi: 10.1186/1471-2393-14-294 25169813 PMC4159505

[25] Mojahed A, Alaidarous N, Kopp M, et al. Prevalence of intimate partner violence among intimate partners during the perinatal period: A narrative literature review. Front Psychiatry 2021;12:601236; doi: 10.3389/fpsyt.2021.601236 33633606 PMC7900188

[26] Chan KL, Lo CKM, Lu Y, et al. Intimate partner violence before pregnancy, during pregnancy, and after childbirth: A new conceptualization highlighting individual changes in violence against pregnant women over time. J Interpers Violence 2022;37(13–14):Np12111–Np12132; doi: 10.1177/0886260521997451 33666122

[27] Peitzmeier SM, Fedina L, Ashwell L, et al. Increases in intimate partner violence during COVID-19: Prevalence and correlates. J Interpers Violence 2022;37(21–22):NP20482–NP20512. 34866451 10.1177/08862605211052586 PMC9014340

[28] Bhuptani PH, Hunter J, Goodwin C, et al. Characterizing intimate partner violence in the United States during the COVID-19 pandemic: A systematic review. Trauma Violence Abuse 2023;24(5):3220–3235. 36321779 10.1177/15248380221126187

[29] Chen X-Y, Lo CKM, Chen Q, et al. Intimate partner violence against women before, during, and after pregnancy: A meta-analysis. Trauma Violence Abuse 2024;25(4):2768–2780. 38265064 10.1177/15248380241226631

[30] Capaldi DM, Knoble NB, Shortt JW, et al. A systematic review of risk factors for intimate partner violence. Partner Abuse 2012;3(2):231–280. 22754606 10.1891/1946-6560.3.2.231 PMC3384540

[31] Cunradi CB, Caetano R, Schafer J. Socioeconomic predictors of intimate partner violence among White, Black, and Hispanic couples in the United States. J Fam Violence 2002;17(4):377–389.

[32] Brownridge DA, Halli SS. Double jeopardy? Violence against immigrant women in Canada. Violence Vict 2002;17(4):455–471; doi: 10.1891/vivi.17.4.455.33680 12353592

[33] Johnson E, Jenssen S, Wernette GT, et al. Web-based intervention to reduce intimate partner violence during perinatal period: A modified protocol in response to the COVID-19 pandemic. Psychiatry Res 2022;317:114895; doi: 10.1016/j.psychres.2022.114895 37406076 PMC9556943

